# Targeting 14-3-3ε-CDC25A interactions to trigger apoptotic cell death in skin cancer

**DOI:** 10.18632/oncotarget.27700

**Published:** 2020-09-01

**Authors:** Thomas R. Holmes, Jenan Al-Matouq, Matti Holmes, Lauren Nicola, Justin C. Rudd, Sándor Lovas, Laura A. Hansen

**Affiliations:** ^1^Creighton University School of Medicine, Department of Biomedical Sciences, Omaha, NE, USA; ^2^Current Address: Northwestern University, Chicago, IL, USA; ^3^Current Address: Mohammed Al-mana College for Medical Science, Dammam, Kingdom of Saudi Arabia

**Keywords:** skin cancer, squamous cell carcinoma, CDC25A, 14-3-3ε, apoptosis

## Abstract

Non-melanoma skin cancer is the most common form of cancer worldwide. We previously documented an anti-apoptotic role for CDC25A in cutaneous squamous cell carcinoma (SCC), an activity dependent on its association with 14-3-3 proteins. We hypothesized that targeting CDC25A-14-3-3ε interactions may be an effective strategy for inducing skin cancer cell apoptosis. Co-immunoprecipitation revealed that CDC25A associated with 14-3-3ε, 14-3-3γ and 14-3-3ζ in SCC cells but not normal keratinocytes. 14-3-3ε and CDC25A activated Akt/BAD/Survivin pro-survival signaling. To target the interaction of 14-3-3ε with CDC25A for cancer therapy, we developed two novel phospho-peptides, pS and pT, corresponding to each of the 14-3-3 binding sites of CDC25A, to specifically interfere with 14-3-3ε binding to CDC25A. Peptides pT (IC_50_ = 22.1 μM), and pS (IC_50_ = 29 μM) induced SCC cell death and blocked 14-3-3ε binding to CDC25A. pS or pT treatment of SCC xenografts increased apoptotic cell death and decreased pro-survival P-Akt (S473) and Survivin, demonstrating the effectiveness of the peptides *in vivo*. These findings lay a framework for the further development of peptides to target 14-3-3ε-CDC25A interactions for skin cancer treatment.

## INTRODUCTION

Skin cancer is the most common malignancy in humans, with squamous cell carcinoma (SCC) representing 20% of cases [1]. SCCs account for the most skin cancer-related deaths, with only a 34% survival rate for metastatic SCC [2]. Most SCC arise from a premalignant precursor called actinic keratosis (AK), which has up to a 20% risk of progression to SCC [3]. Treatment options for AK are mostly limited to surgical removal or treatment with topical 5-fluorouracil, which can result in undesirable scarring or adverse side effects [4]. It is clear that more work is needed to decipher the mechanisms of skin cancer development and progression in order to uncover novel therapeutic treatments to block progression of AK.

We previously documented how the phosphatase CDC25A is overexpressed and mislocalized to the cytoplasm in SCC where it inhibits apoptosis [5]. The anti-apoptotic activity of CDC25A was reliant upon its ability to associate with the 14-3-3 family of signaling proteins through its 14-3-3 binding region around residues phosphoserine-178 and phosphothreonine-507 [6]. 14-3-3 proteins associate with many functionally diverse molecules through conserved either phosphoserine or phosphothreonine peptide motifs on target proteins, thus affecting apoptosis, proliferation, metabolism and migration [7–9]. Association of CDC25A with 14-3-3 proteins also occurs in response to cell cycle checkpoint activation following ultraviolet (UV) radiation-induced DNA damage [6, 10, 11].

14-3-3ε, a member of the 14-3-3 family, has been implicated as a tumor promoter in various cancers where it can increase proliferation, inhibit cell death or promote invasion [12–22]. In cutaneous SCC we showed that, similar to CDC25A, 14-3-3ε is enriched in the cytoplasm of SCC cells where it inhibits apoptosis through promotion of Akt/BAD/Survivin pro-survival signaling. Association of CDC25A with 14-3-3ε inhibits the ability of CDC25A to promote cell cycle progression in neuronal and HeLa cells [6, 23]. Specifically, in neuronal cells, release of CDC25A from 14-3-3ε binding increases the phosphatase activity of CDC25A and increases neuronal cell death [23]. However, whether 14-3-3ε associates with CDC25A in skin cancer cells, and what effect targeting this interaction may have on SCC cell viability has not been previously reported.

Here, we investigate CDC25A binding to 14-3-3 isoforms, revealing its interaction with 14-3-3ε, -γ and -ζ. CDC25A and 14-3-3ε similarly activated a Akt/BAD/Survivin pro-survival signaling pathway in SCC cells and suppressed cell death. From these data and our published work we hypothesized that interfering with 14-3-3ε binding to CDC25A would be a useful strategy for killing skin cancer cells. We characterized two phospho-peptide fragments of CDC25A phospho-Ser178 (pS) and phospho-Thr507 (pT), which are part of the region of CDC25A’s 14-3-3ε binding sites, in order to disrupt 14-3-3ε-CDC25A binding. Both peptides reduced 14-3-3ε and CDC25A interactions in SCC cells and also increased apoptosis. Xenograft tumors treated with either pS or pT displayed increased apoptosis, and reduced P-Akt (S473) and Survivin compared to vehicle-treated tumors. These data present a strategy for targeting the 14-3-3ε-CDC25A interaction in SCC cells to induce skin cancer cell death.

## RESULTS

### CDC25A associated with 14-3-3ε, 14-3-3γ and 14-3-3ζ in SCC cells

**Figure 1:**
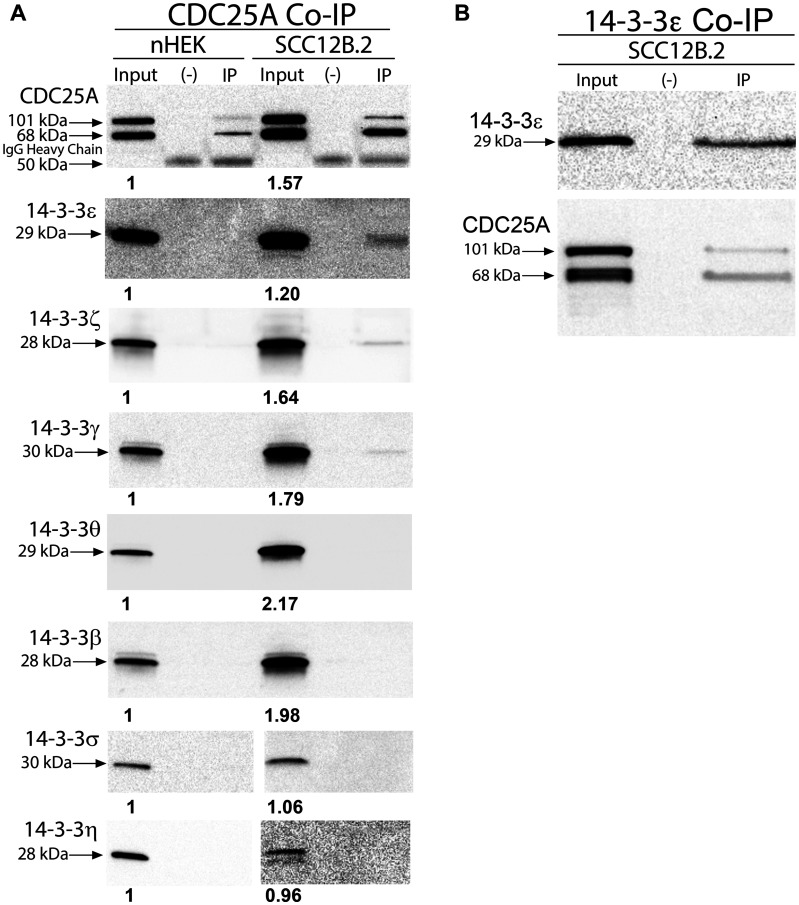
CDC25A associated with anti-apoptotic 14-3-3ε, γ and ζ in SCC cells. (**A**) Co-immunoprecipitation of antibody-CDC25A complexes from 100 mg of nHEK (left) or SCC12B.2 (right) cells followed by immunoblotting for 14-3-3 family members. (**B**) Co-immunoprecipitation of antibody-14-3-3ε complexes from 100 mg of SCC12B.2 cells followed by immunoblotting for CDC25A. Blots are representative of at least 2 separate experiments. Negative (–) control using equal number of cells incubated with IgG isotype control antibody. Bold numbers below bands (input lanes) represent densitometry normalized to nHEK.

In order to develop a strategy for targeting CDC25A binding to 14-3-3ε, we first needed to determine which 14-3-3 isoforms are expressed in skin cancer and also associate with CDC25A in skin cancer cells. Immunoblotting for 14-3-3 isoforms revealed increased levels of 14-3-3ε, -ζ, -γ, -θ and -β in SCC cells compared to normal keratinocytes (Figure 1A, Supplementary Figure 1A). Additionally, immunohistochemistry showed that 14-3-3ε and -ζ were increased in human SCC compared to skin [24], whereas levels of 14-3-3σ were decreased in SCC compared to normal skin and 14-3-3η was relocalized to the cytoplasm in SCC (Supplementary Figure 1B). Immunoprecipitation of CDC25A from SCC12B.2 cells revealed its association with 14- 3-3ε, -ζ, and -γ (Figure 1A). Association of CDC25A with 14-3-3 isoforms was not detectable in nHEK cells (Figure 1A). Immunoprecipitation of 14-3-3ε from SCC12B.2 cells revealed its association with CDC25A, confirming their interaction (Figure 1B). These findings led us to further examine CDC25A and 14-3-3ε signaling in SCC cells.

### CDC25A and 14-3-3ε promoted pro-survival signaling in SCC cells

**Figure 2:**
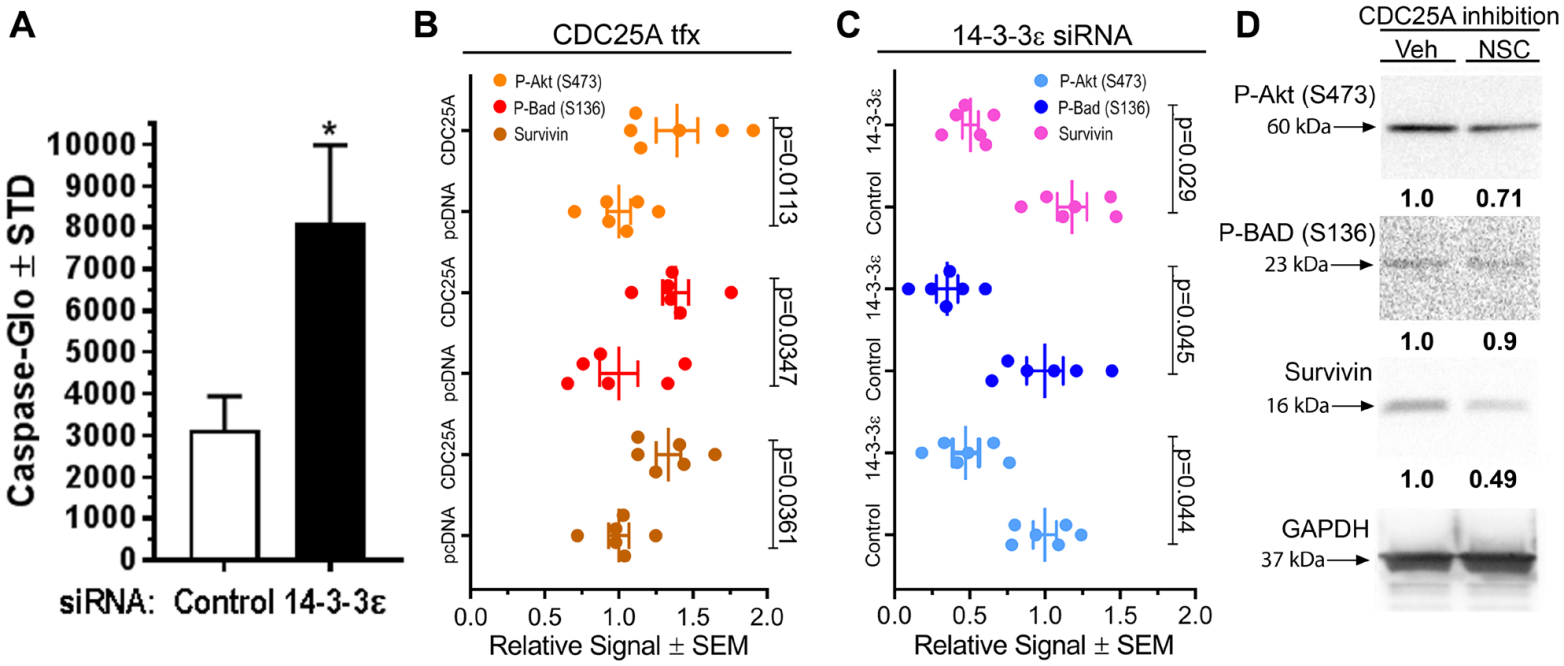
CDC25A and 14-3-3ε regulated pro-survival signals in SCC cells. (**A**) Caspase-Glo assay was performed on SCC12B.2 cells 24 h after transfection of control or *14-3-3ε* targeting siRNA (*N* = 3). Representative of several experiments performed. ^*^Indicates a significant difference compared to control, where *p* = 0.013 using a Student’s *t*-test. (**B**–**C**) Apoptosis antibody array for expression of apoptosis signaling proteins from protein lysates of SCC12B.2 cells 48 h after CDC25A or control pcDNA transfection (B) or *14-3-3ε* silencing or control siRNA treatment (C). The antibody array represents 21 targets involved in apoptotic signaling, including tubulin as a control. Densitometry analysis for P-Akt (S473), P-BAD (S136) and Survivin from 6 total dots (3 membranes) normalized to tubulin is shown. Significance was obtained by performing a one-way ANOVA followed by Dunnett’s post-hoc test, *P* ≤ 0.05. (**D**) SCC12B.2 cells were treated with vehicle or CDC25A inhibitor NSC663284 (NSC) (5 μM) for 24 h followed by analysis of indicated antibodies by immunoblot. GAPDH is the loading control. Immunoblots and densitometry are representative of three independent experiments.

We previously documented an anti-apoptotic role for CDC25A in SCC that is dependent on its ability to interaction with 14-3-3 [5]. Because CDC25A strongly associated with 14-3-3ε, we hypothesized that 14-3-3ε may inhibit cell death through a common mechanism with CDC25A. To test the effect of 14-3-3ε on apoptosis in SCC cells, *14-3-3ε* was silenced in SCC12B.2 cells, revealing significantly increased apoptosis in a Caspase activity assay (Figure 2A). An antibody array was deployed using lysates from SCC12B.2 cells transfected with *14-3-3ε* siRNA or control siRNA, or empty vector DNA or *CDC25A* plasmid DNA. Forced expression of *CDC25A* increased P-Akt (S473), inhibitory phosphorylation of BAD (S136) and total Survivin (Figure 2B). Conversely, silencing *14-3-3ε* decreased P-Akt (S473), P-BAD (S136) and Survivin levels (Figure 2C). Inhibition of CDC25A activity decreased P-Akt (S473) and Survivin in SCC cells with a slight decrease in P-BAD (S136) levels (Figure 2D). From these data we concluded that 14-3-3ε and CDC25A similarly activate Akt/BAD/ Survivin pro-survival signaling in SCC cells to suppress cell death.

### Peptide design and molecular dynamic (MD) simulations

**Figure 3:**
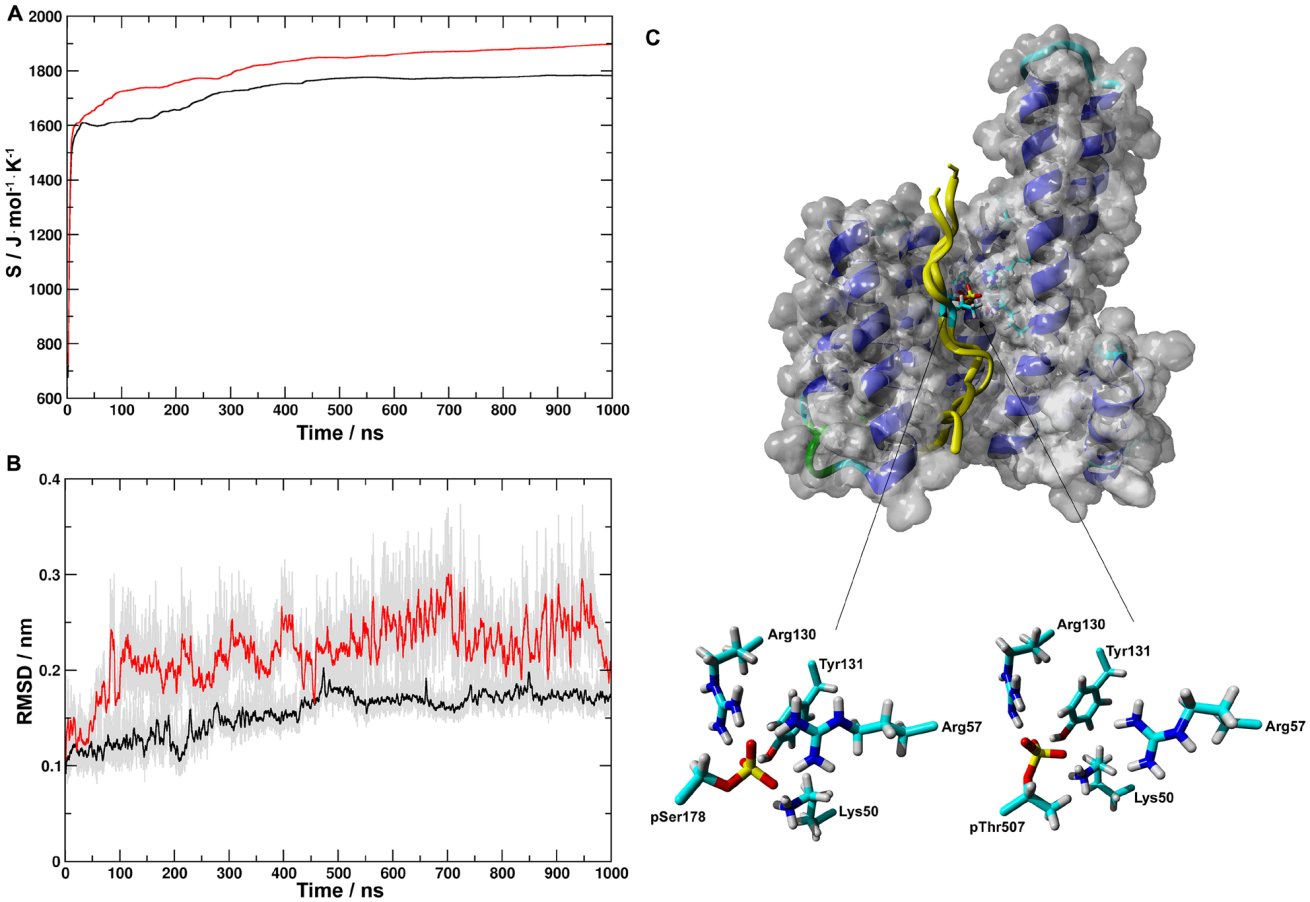
Molecular dynamics simulations of structures of pS- and pT-14-3-3ε complexes. (**A**) Evolution of configurational entropies for pS- (black) and pT- (red) 14-3-3ε complexes; for both systems after an initial increase in the values of configurational entropy between 0 ns and ~20 ns, an extended period of gradual increasing occurred between 50 ns and 500 ns after which an equilibration being reached. (**B**) Running averages at 100 ps of RMSDs of the Cα-atoms for pS-(black) and pT- (red) 14-3-3ε complexes indicated the similar trend in reaching the equilibrium during simulations. For both simulations the total samplings in gray color. (**C**) (top) Representative structures of the most populated clusters for the last 500 ns of the MD simulations are shown in van der Waals surface-ribbon and tube representation of 14-3-3ε and both pS and pT peptides, respectively; (bottom) pSer178 and pThr507 of pS and pT, respectively, and Lys50, Arg59, Arg130 and Tyr131 of 14-3-3ε are indicated by sticks. During simulations both peptides stayed bound in the binding groove of 14-3-3ε, showing strong interactions between the peptides and 14-3-3ε.

The -Arg-Gln-Asn-pSer-Ala-Pro- sequence around pSer178 of CDC25A corresponds to the binding motif 1 (-RSXpSXP-) of 14-3-3 client proteins [8]. The -Lys-Ser-Arg-pThr-Trp-Ala- sequence at pThr507 does not fit either binding motifs. Nevertheless, Chen and colleagues [6] proposed that it still can be part of the 14-3-3ε binding region of CDC25A. Therefore, the 173-186 (pS peptide) and 501-515 (pT peptide) fragments of human CDC25A were complexed with the X-ray structure of 14-3-3ε (PDB i.d. 3ual) [25]. Both peptides were N-terminally acetyl- and C-terminally amide-protected to preserve the electronic structure of the backbone as in CDC25A. The stability of the peptide-14-3-3ε complexes were studied in 1000 ns MD simulations. During simulations, after initial minimal rearrangements, both peptides stayed in the binding groove of 14-3-3ε. Configurational entropies for pS- and pT-14-3-3ε complexes (Figure 3A) for both systems initially increased in values between 0 ns and ~20 ns and followed by an extended period of gradual increase between 50 ns and 500 ns after which an equilibrium was reached. Changes in Cα-atom RMSD showed similar trends (Figure 3B). Trajectories of the last 500 ns of simulations were submitted to cluster analysis. For the pS-14-3-3ε simulation two clusters were identified of which the largest cluster contained 98% of the sampled configurations. For the pT-14-3-3ε simulation 5 clusters were identified of which the largest cluster contained 95.6% of the sampled configurations. Representative structures of the most populated clusters are shown in Figure 3C. Both the phospho-Ser and phospho-Thr residues interacted through hydrogen bonds and/or salt bridges with Lys50, Arg57, Arg130 and Tyr131 of 14-3-3ε (Figure 3C).

### pS and pT peptides blocked 14-3-3ε-CDC25A binding, reduced cell viability and increased apoptosis in SCC cells

**Figure 4:**
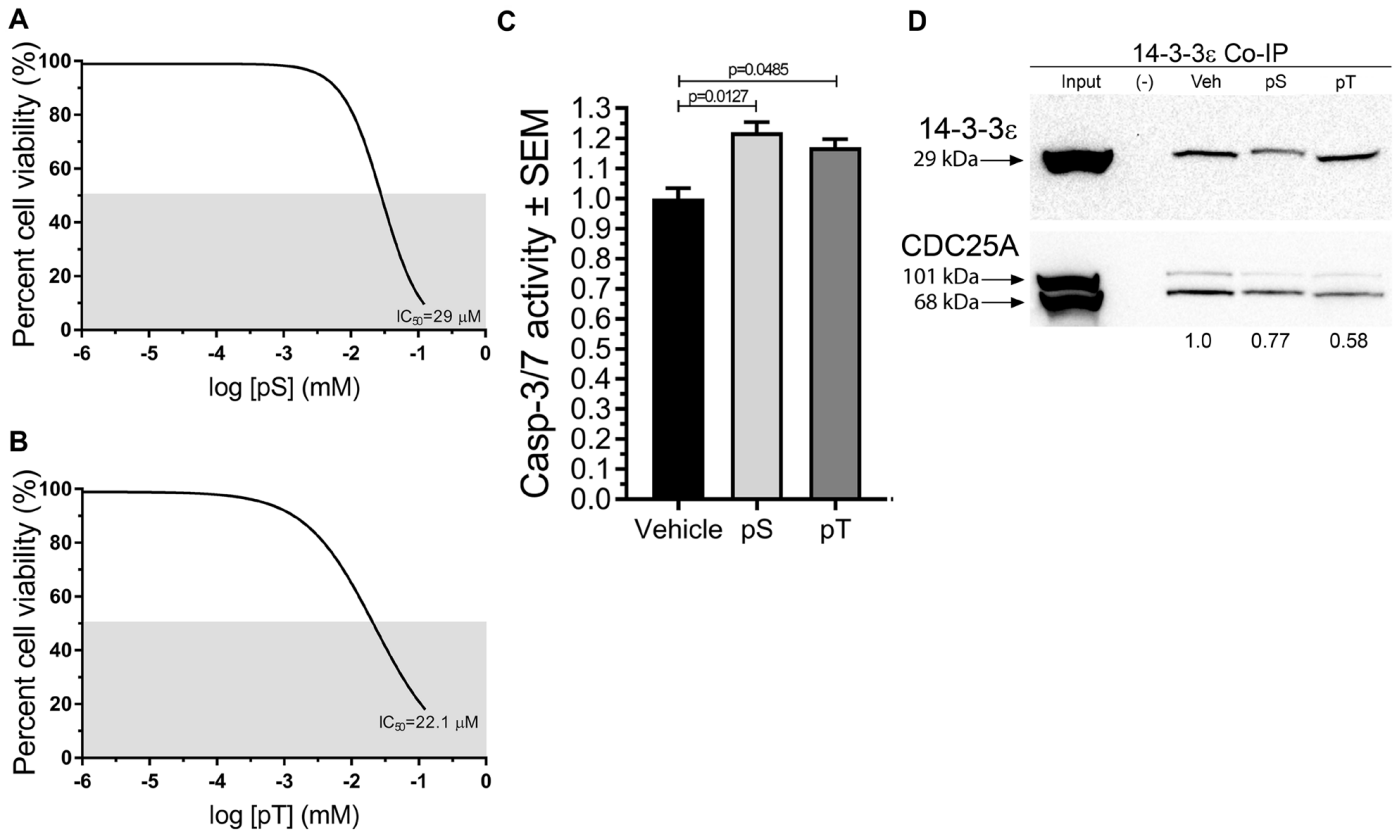
Synthetic fragments pS and pT of CDC25A disrupted CDC25A binding to 14-3-3ε, reduced cell viability, and increased apoptosis in SCC cells. (**A**, **B**) Neutral Red Cell Viability Assays were employed to determine the IC_50_ of pS and pT in SCC12B.2 cells using increasing concentrations of peptide ranging from 0 to 75 μM; gray area indicates 50% survival threshold; graphs represent the average IC_50_ from 3 biological replicates with each treatment condition having 4 technical replicates (*N* = 3). (**C**) Apoptosis was assessed in SCC12B.2 cells treated with vehicle, pS (30 μM) or pT (20 μM) for 24 h followed by a Caspase-3/7 Glo assay (*N* = 5). (**D**) Immunoblots of co-immunoprecipitated 14-3-3ε protein from SCC12B.2 cells treated with vehicle (Tris buffer, pH, 7.5), pS (30 μM) or pT (20 μM) for 48 h; Negative (–) control using equal number of cells incubated with IgG isotype control antibody; images and densitometry (normalized to vehicle lane) are representative of three separate experiments. Statistical significance was obtained by performing a one-way ANOVA followed by Dunnett’s post-hoc test, *P* ≤ 0.05.

On the basis of MD simulation results we hypothesized that blocking interactions between CDC25A and 14-3-3ε using synthetic phospho-peptide fragments (pS and pT) from the putative binding sites (Ser178 and Thr507) would decrease SCC cell survival. Therefore, SCC12B.2 cells were treated with increasing concentrations of either pS or pT for 96 h to determine the half maximal inhibitory concentration (IC_50_) of each peptide using a neutral red cell viability assay [26]. Both pS and pT decreased SCC cell viability with IC_50_ values of 29 μM and 22.6 μM, respectively (Figure 4A, 4B), and significantly increased apoptosis in SCC cells (Figure 4C). Similarly, a pan inhibitor of 14-3-3-client interactions, R18, was able to kill SCC cells with no effect on normal keratinocytes (data not shown).

Next, we wanted to decipher whether pS and pT were able to interfere with interaction of 14-3-3ε with CDC25A. Immunoprecipitation of 14-3-3ε revealed a decrease in CDC25A on immunoblots from SCC cells treated with pS or pT when compared to 14-3-3ε immunoprecipitations from vehicle treated cells (Figure 4D). Of note, there was consistently less 14-3-3ε precipitated from pS treated samples despite equal numbers of cells being used for each reaction.

### Targeting 14-3-3ε-CDC25A binding increased apoptosis *in vivo*

**Figure 5:**
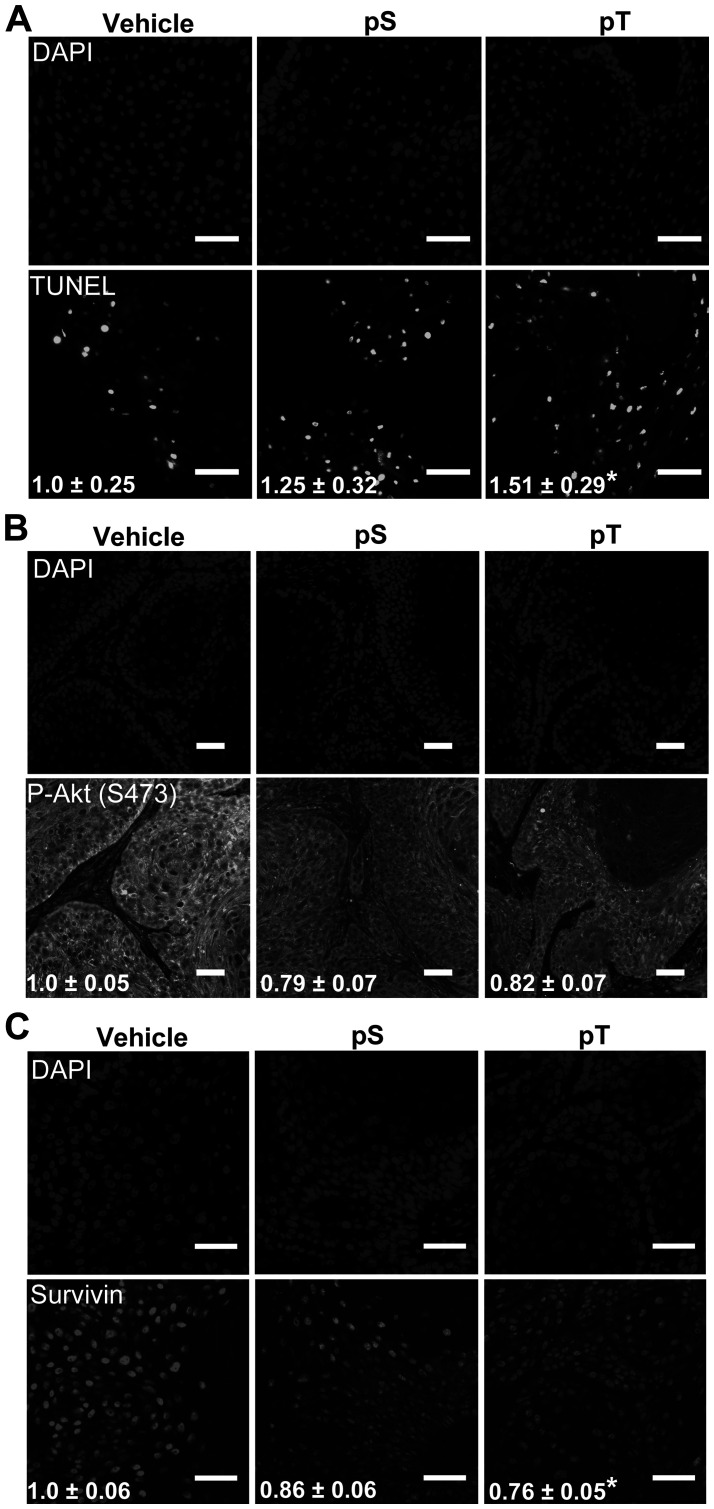
Targeting 14-3-3ε-CDC25A binding increases apoptosis *in vivo*. (**A**) TUNEL labeling to detect apoptotic nuclei (bottom) was performed on whole tumor sections from vehicle, pS or pT-treated mouse xenografts with DAPI-labeled nuclei (top) (scale bar = 50 μm); the average number of apoptotic cells per μm^2^ ± SEM, indicated in white font, were obtained by counting TUNEL positive cells on whole tumor sections using Olympus VS120 analysis software and normalizing to the area of the tumor section; images are representative of tumors from 3 mice (*N* = 3). (**B**, **C**) Immunofluorescence for P-Akt (S473) (B) or Survivin (C) (bottom) was performed on whole tumor sections from vehicle, pS or pT (*N* = 3) treated tumors with DAPI identification of nuclei (top) (Scale bar = 50 μm); white font indicates the average FITC intensity ± SEM of the whole tumor section from each mouse (*N* = 3). Significance determined using a two-tailed Student’s *t*-test, *P* ≤ 0.05.

To evaluate whether pS or pT could kill SCC cells *in vivo*, immunocompromised NCG mice were injected with 5 × 10^5^ SCC13 cells to establish xenograft tumors. After intratumoral injection of vehicle, pS or pT for two days, TUNEL positivity was increased by 25% and 51% in pS and pT treated tumors, respectively, when compared to vehicle treated tumors (Figure 5A). Additionally, immunofluorescence revealed a decrease in Survivin levels and active Akt assayed using a P-Akt (S473) antibody, in both pS and pT treated tumors compared to control (Figure 5B, 5C). These data displayed the efficacy of our peptides for targeting 14-3-3ε-CDC25A binding *in vivo*.

## DISCUSSION

In summary, we showed that CDC25A associated with 14-3-3ε, 14-3-3γ and 14-3-3ζ in SCC cells. Forced expression of *CDC25A* increased Akt activity, inhibition of BAD and Survivin levels in SCC cells, while inhibition of CDC25A or silencing of *14-3-3ε* had the opposite effect and increased apoptosis. Two synthetic peptide fragments of CDC25A pS and pT, corresponding to 14-3-3 binding sites, were characterized *in vitro* and *in vivo*. Disruption of 14-3-3ε-CDC25A binding with pS or pT reduced SCC cell survival and anti-apoptotic signaling, and increased apoptosis both *in vitro* and *in vivo*. These data suggest that inhibition of 14-3-3ε binding to CDC25A may be a successful strategy for skin cancer treatment.

We previously showed that CDC25A inhibits apoptosis in SCC, a function that is closely tied to its cytoplasmic localization [5, 27]. Herein we demonstrate that this effect involves Akt, BAD and Survivin signaling. In rat cells, CD25A also inhibited apoptosis by inducing an increase in active P-Akt (S473) [28]. Fuhrmann and colleagues also found that CDC25A and Akt precipitated with Raf1, which is known to be directly activated by the removal of inhibitory phosphate groups by CDC25A [29, 30]. The authors postulated that CDC25A could be indirectly increasing P-Akt (S473) by directly activating Raf1 [28]. Interestingly, CDC25A interacts with Raf-1 via 14-3-3 protein scaffolding [31]. It is plausible that this same pathway is occurring in our SCC cells. More work is needed to determine the effect of CDC25A on Raf-1 phosphorylation and how Raf-1 may impact Akt activation in skin cancer.

We showed that CDC25A interacts with 14-3-3ε, -γ and -ζ in human SCC cells, but does not show a detectable association with any 14-3-3 isoform in normal skin keratinocytes. CDC25A has previously been shown to interact with 14-3-3ε, β and γ where they can sequester CDC25A into the cytoplasm [6, 31–33]. Specifically, Kasahara *et al.* showed that, during ultraviolet (UV)-induced DNA damage 14-3-3γ delivers CDC25A to Chk1 leading to phosphorylation of CDC25A and subsequent ubiquitin mediated degradation [32]. We show an interaction between CDC25A and 14-3-3ε, -ζ and -γ in SCC cells. However, despite the strong expression of 14-3-3θ and 14-3-3β in SCC cells compared to normal keratinocytes, we did not see any association of these two isoforms with CDC25A. Association of CDC25A with 14-3-3ε is in agreement with the results of others where 14-3-3ε is the most frequent CDC25A binding partner of all the 14-3-3 isoforms [6, 31, 33].

In HeLa cells 14-3-3ε can stabilize CDC25A, while at the same time blocking its ability to activate cyclin-dependent kinase 1 (CDK1) to induce cell cycle progression [33]. Data from our laboratory, in contrast, revealed that CDC25A regulates 14-3-3ε expression. Mouse skin and skin tumors lacking *CDC25A* expression show decreased levels of 14-3-3ε protein and *14-3-3ε* transcripts compared to wild type skin and tumors (not shown). The difference observed between HeLa and keratinocyte cells may be linked to differing localization patterns of CDC25A and 14-3-3ε in the cell lines. Modes of *CDC25A* and *14-3-3ε* transcriptional regulation may also differ between cell types. Understanding how CDC25A can regulate 14-3-3ε expression, whether it can affect the expression of other 14-3-3 isoforms, and how this affects the skin’s response to UV-induced DNA damage and tumorigenesis may provide more insight into the role of CDC25A and 14-3-3ε in skin cancer development.

We have shown that in cutaneous SCC, 14-3-3 associates with CDC25A in the cytoplasm, a location that prevented CDC25A from promoting cell proliferation in the nucleus, but allowed it to inhibit apoptosis in the cytoplasm [5]. Others have similarly shown a close relationship between cytoplasmic CDC25A and promotion of cell survival [27, 30]. These data provide insight into how 14-3-3 binding to CDC25A is rewired in cancer cells to shift CDC25A away from its more common, cell cycle role.

In conclusion, we have demonstrated that CDC25A associated with 14-3-3ε, 14-3-3γ and 14-3-3ζ in SCC cells. CDC25A and 14-3-3ε inhibited apoptosis by promoting the activation of Akt, inhibition of BAD and an increase in Survivin, the same pathway that we have shown to be regulated by 14-3-3ε. Also, to the best of our knowledge, this is the first time that P-CDC25A (Ser178) and P-CDC25A (Ser507) peptide fragments have been shown to directly interact with 14-3-3ε in a cellular environment. Treatment of SCC cells with the peptide fragments reduced the interaction of 14-3-3ε with CDC25A, decreased SCC cell viability and increased apoptosis both *in vitro* and *in vivo*. In all, we have demonstrated the potential of blocking CDC25A binding to 14-3-3ε in targeting skin cancer cells.

## MATERIALS AND METHODS

### Cell culture

Previously characterized human carcinoma cell lines (SCC12B.2 and SCC13) [34, 35] were maintained in Dulbecco’s modified Eagle’s medium (DMEM) (Invitrogen, Carlsbad, CA, USA) supplemented with 1% penicillin (10,000 U/ml)-streptomycin (10,000 μg/ml) (PenStrep) (Invitrogen) and 10% fetal bovine serum (Gemini Bio-Products, Sacramento, CA, USA) at 37°C and 5% CO_2_. Primary normal human epidermal keratinocytes (nHEK) (Thermo Fisher Scientific, Waltham, MA, USA) isolated from neonatal foreskin were maintained in EpiLife medium (Invitrogen) supplemented with human keratinocyte growth supplement (HKGS) and 1% PenStrep. nHEK cells were never passaged more than twice before experiments. Cells were treated with CDC25A inhibitor NSC663284 (Tocris Bioscience, Bristol, United Kingdom) suspended in DMSO, transfected with wild type CDC25A [6] or control plasmid using Lipofectamine/Plus transfection reagents (Life Technologies, Carlsbad, CA, USA) or transfected with siRNA targeting 14-3-3ε or control siRNA (Santa Cruz Biotechnology, Dallas, TX, USA) using siQuest transfection reagents (Mirus Bio, Madison, WI, USA).

### Peptide structure and molecular dynamics simulations

The pS and pT peptides consisting of the 173-186 and 501-515 fragments of human CDC25A, respectively, were generated by the YASARA program [36]. Peptide pS contains the phospho-Ser178 (pSer) and pT has the phospho-Thr507 (pThr) residue. Residues of the existing peptide in the X-ray structure of 14-3-3ε (PDB i.d. 3ual) [25] were mutated to the corresponding CDC25A fragments. Both peptides were N-terminally acetyl- and C-terminally amide-protected to preserve the electronic structure of the backbone as in CDC25A. The resultant structure was energy-minimized in gas phase using two steps: 1) The atoms of 14-3-3ε were frozen and the ligand peptide was relaxed and 2) in a subsequent step the whole system was relaxed. Stability of the prepared peptide-14-3-3ε complexes was determined by molecular dynamics (MD) simulations using the GROMACS-2016 package [37] and the CHARMM36m force field [38].

The structures of the 14-3-3ε-peptide complexes were solvated in a dodecahedron with TIP3P water molecules [39] and 0.15 M sodium chloride. The system was subjected to 1000 steps steepest descent energy minimization and then to 1 ns constant number of molecules, volume and temperature (NVT) simulation at 300 K so that the position of the protein-peptide complex was constrained at the center of the dodecahedron with a force constant of 1000 kJ·mol^-1^. The system was then equilibrated during 10 ns unrestrained constant number of molecules, pressure and temperature (NPT) simulation at 1 bar pressure and 300 K temperature. The integration step was 2 fs, the LINCS algorithm [40] was used to constrain all bonds to their correct length with a warning angle of 30°. The peptide and the solvent with ions were coupled to separate temperature baths with a relaxation constant of 0.1 ps. The system was coupled to constant pressure using the method of Berendsen with a relaxation constant of 1.0 ps and 4.5 × 10^−5^ bar^-1^ isothermal compressibility [41]. The temperature was controlled by the stochastic velocity-rescaling method of Bussi [42]. The long-range electrostatic interactions were calculated using the PME method with 1.2 nm cutoff distance applying the Verlet scheme and 0.15 nm Fourier spacing. For the calculations of van der Waals interactions the short-range and long-range cutoffs, respectively, were 1.0 and 1.2 nm using the force-switch modifier. Finally, 1000 ns NPT simulation was performed at 300 K and 1 bar pressure. The parameters were the same as during equilibration, except that the temperature was kept constant by using leap-frog stochastic dynamics integrator with τ_T_ of 2 ps and the system was coupled to 1 bar Parrinello-Rahman barostat [43].

### Trajectory analysis

Configurational entropy was calculated to determine if thermodynamic equilibrium was reached. The covariance matrix for the Cα-atoms was calculated using the *covar* module of GROMACS, the eigenvectors corresponding to the 150 highest eigenvalues were used to calculate the backbone configurational entropy [44]. Stability of the protein-peptide complex was determined by calculating the root-mean-square deviations (RMSD) of the Cα-atoms. Representative structures of the simulation were determined by using the GROMOS method of clustering [45] with Cα-atoms RMSD cutoff of 0.2 nm using structures sampled in 100 ps intervals.

### Peptide production

Peptides were synthesized and purified to greater than 95% purity by Bachem (Bachem Americas Inc., Torrance, CA, USA) and EZBiolabs (EZBiolabs, Carmel, IN, USA).

### Immunofluorescence

Immunostaining was performed using antibodies recognizing P-Akt (S473) (Thermo Fisher) and Survivin (Cell Signaling, Danvers, MA, USA), with alexa-fluor488-conjugated secondary antibody (IgG rabbit) (Invitrogen) with 4′,6-diamidino-2-phenylindole (DAPI) (Vector Labs, Burlingame, CA, USA) to identify the nuclei. Slides were digitalized and analyzed using an Olympus VS120 Slide Scanner (Olympus, Shinjuku, Tokyo, JPN).

### Immunohistochemistry

Immunohistochemistry was performed using antibodies to detect 14-3-3ε, β, η, γ, σ, θ or ζ (Cell Signaling for all), with biotinylated secondary antibody (IgG rabbit) (Jackson Laboratories, Bar Harbor, ME, USA). HRP-streptavidin (Vector Labs, Burlingame, CA, USA) and 3,3 diaminobenzidine (DAB) substrate kit (BD Biosciences, Franklin Lakes, NJ, USA) were used for signal development.

### Co-immunoprecipitation

Co-Immunoprecipitation (Co-IP) was performed using the Dynabeads Protein G Immunoprecipitation kit (Life Technologies, Carlsbad, CA, USA). Beads were coupled to antibodies targeting 14-3-3ε (Fisher Scientific, Waltham, MA, USA) or CDC25A (Santa Cruz Biotechnology, Dallas, TX, USA) at a ratio of 7 μg of antibody for every 1 mg of beads according to the manufacturers protocol. SCC12B.2 cells were treated with vehicle (20 mM Tris buffer, pH 7.5) or fresh peptide daily at the IC_50_ concentration for 48 h in Opti-MEM I Reduced Serum Medium (Invitrogen) and collected by trypsinization. One hundred mg of cells were lysed in 1× IP buffer (provided by supplier) supplemented with 100 mM sodium chloride, 2 mM dithiothreitol, 1 mM magnesium chloride and protease/phosphatase inhibitors. Immunoprecipitation reactions were carried out using 1.5 mg of antibody-coupled beads incubated with 100 mg of cell lysate at 4°C for 30 minutes. Negative controls were immunoprecipitation reactions using beads coupled to IgG isotype control antibody (rabbit), and whole lysate from immunoprecipitation input loaded directly onto a gel was used as a positive control.

### Immunoblotting

Protein was collected by either standard whole-cell lysis using ice-cold radioimmunoprecipitation assay (RIPA) lysis buffer (10 mM NaPO_4_, 150 mM NaCl, 1% triton X) supplemented with protease (Roche Life Scientific) and phosphatase inhibitors (1 mM NaF, 1 mM Na_3_VO_4_) or by nuclear/cytosolic extraction using the NE-PER Nuclear and Cytoplasmic Protein Extraction Reagent Kit according to the manufacturer’s protocol (Pierce, Rockford, IL, USA). Tissue protein was extracted by using a tissue grinder to lyse cells in 100 μL of RIPA lysis buffer for every 1 mg of tissue. Samples were centrifuged for 5 minutes to remove depleted tissue debris. Protein concentration was assessed with the Bradford assay. Protein lysates (30 μg/lane) were resolved on a 10% SDS-PAGE denaturing gel, transferred to a nitrocellulose membrane and blocked in 5% non-fat dry milk in 1× Tris buffered saline supplemented with 0.01% Tween 20. Immunoblotting was performed by incubating membranes overnight at 4°C using antibodies to detect 14-3-3ε, β, η, γ, σ, θ or ζ (Cell Signaling for all), CDC25A (Santa Cruz Biotechnology), P-Akt (S473) (Cell Signaling), total Akt (Cell Signaling), P-Bad (S136) (Cell Signaling), total BAD (Cell Signaling) Survivin (Cell Signaling) or GAPDH (Cell Signaling). Bands were detected using horseradish peroxidase-conjugated secondary antibodies (IgG mouse or rabbit) (Cell Signaling) and visualized using western lightning plus chemiluminescent reagent (MidSci, Valley Park, MO, USA). The signal was read, and densitometry was performed using a Chemidoc XRS Molecular Imager and accompanying software (Bio-Rad Laboratories, Hercules, CA, USA).

### Apoptosis antibody array

Protein was extracted from SCC12B.2 cells using lysis buffer supplemented with protease and phosphatase inhibitors. SCC12B.2 cells were transfected with empty vector DNA or wild-type CDC25A [6] for 48 h, or control (Santa Cruz Biotechnology) or 14-3-3ε siRNA for 48 h (Dharmacon). Array membranes (Cell Signaling) containing antibodies against 19 pro- and anti-apoptotic proteins were incubated with whole-cell lysates overnight at 4°C according to the manufacturer’s instructions. Immunoreactive dots were visualized using western lightning plus chemiluminescent detection system (MidSci) to generate a signal on a Chemidoc XRS Molecular Imager (Bio-Rad Laboratories).

### Mouse tumor xenografts

Groups of male NCG mice (*NOD CRISPR Prkdc Il2r gamma/NjuCrl*) (Charles River, Malvern, PA, USA) were injected s. c. with 5 × 10^5^ human SCC13 cells suspended in 1:1 mixture of 1× PBS (pH = 7.5): Matrigel (Corning, Corning, NY, USA). Once the tumors reached 150 mm^3^ in volume they were treated intratumorally with either vehicle (1× sterile PBS, pH 7.5) (*N* = 3), 2.5 nmol/50 μL of pS (500 nM) or 2.5 nmol/50 μL pT (500 nM) (*N* = 3 for pS and pT) daily for 2 days and then the mice were euthanized 24 h after the second treatment. Tumors were fixed overnight in 10% neutral buffered formalin (Thermo Fisher) and then switched to 70% ethanol the following day before embedding and sectioning. All procedures were performed in accordance with the guidance of the Creighton University Institutional Animal Care and Use Committee.

### TUNEL assay

TUNEL (Terminal deoxynucleotidyl transferase dUTP nick end labeling) assays were carried out using the Promega DeadEnd Fluorometric TUNEL system in accordance with the manufacturer’s instructions (Promega Corporation, Madison, WI, USA). Cells were treated with DAPI to locate the nuclei within cells. Slides were scanned and analyzed using an Olympus VS120 Slide Scanner (Olympus).

### Caspase-3/7 Glo assay

Apoptosis was assessed using a Caspase-3/7 Glo assay (Promega) in a 96-well white-bottom plate (BRANDplates, Wertheim, Germany). Cells were seeded at a density of 8 × 10^3^ cells/well and Caspase-3/7 glo was performed 24 h post-treatment according to the manufacturer’s instructions and read using a Cytation 5 multi-mode plate reader (BioTek, Instruments, Winooski, VT, USA).

### Neutral red cell viability assay

To determine the half maximal inhibitory concentration (IC_50_) for pS and pT in SCC12B.2 cells, cells were plated in a 96-well plate at a density of 6 × 10^3^ cells/well. Once cells reached 50–60% confluency they were treated for 4 consecutive days with increasing concentrations of pS and pT peptides (0–75 μM in 20 mM Tris buffer pH, 7.5) diluted in Opti MEM media (no additions) (Invitrogen). The peptides were resuspended fresh daily in 20 mM Tris buffer (pH, 7.5). The Neutral Red Cell Viability assay was previously described in [37]. Absorbance was measured using a Cytation 5 multi-mode plate reader (BioTek). The experiment was repeated three separate times with 4 replicate wells in each experiment to obtain an average IC_50_ value using GraphPad Prism Software (GraphPad Software Incorporated, La Jolla, CA, USA).

### Statistical analysis

Significance was determined using one-way ANOVA with the Dunnett’s *post-hoc* test for comparison of more than two groups, where *P* ≤ 0.05. Comparisons between two groups were performed using a two-tailed Student’s *t*-test, where *P* ≤ 0.05.

## SUPPLEMENTARY MATERIALS



## Abbreviations

AK: actinic keratosis
ANOVA: analysis of variance
CDC25A: cell division cycle 25A
MD: molecular dynamics
MT: mutant
pS: phospho-Serine
pT: phospho-Threonine
SCC: squamous cell carcinoma
WT: wild type
