# Supplementary material for: Targeting 14-3-3ε-CDC25A interactions to trigger apoptotic cell death in skin cancer
Source: Oncotarget. 2020 Sep 1;11(35):3267–78. doi: 10.18632/oncotarget.27700 (PMC7476737; doi:10.18632/oncotarget.27700)
Supplement: Supplementary file 1 [file oncotarget-11-3267-s001.pdf]

Targeting 14-3-3ε-CDC25A interactions to trigger apoptotic cell death in skin cancer

SUPPLEMENTARY MATERIALS

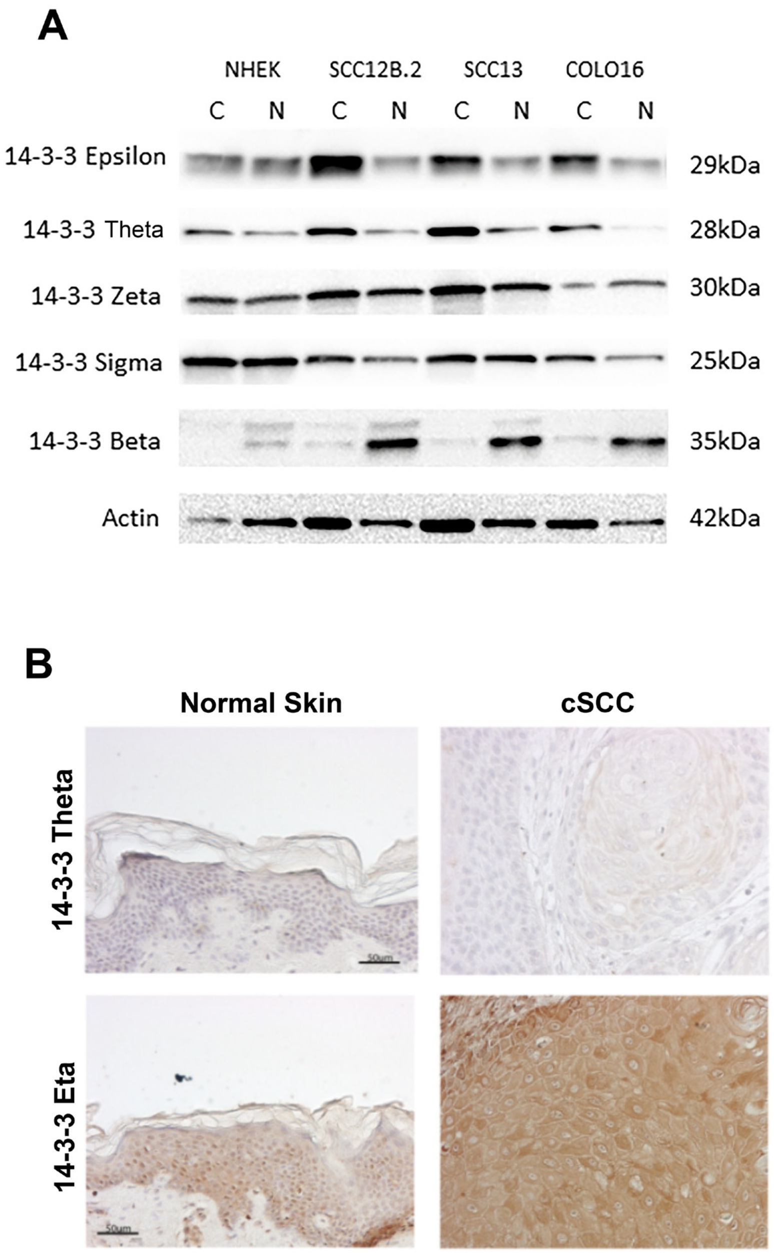

Supplementary Figure 1: Expression patterns of 14-3-3 isoforms in human SCC cell lines, normal human skin and human cSCCs. (**A**) Nuclear and cytoplasmic protein was extracted from normal human epidermal keratinocytes (NHEK) and three human SCC cell lines; SCC12B.2, SCC13 and COLO16. Analysis of indicated 14-3-3 isoform expression was performed by immunoblot. (**B**) Immunohistochemistry was performed on normal human skin and human SCC to detect 14-3-3 isoform expression.
